# Microbiome influencers of checkpoint blockade–associated toxicity

**DOI:** 10.1084/jem.20220948

**Published:** 2023-01-09

**Authors:** Yinghong Wang, Robert R. Jenq, Jennifer A. Wargo, Stephanie S. Watowich

**Affiliations:** 1 Department of Gastroenterology, Hepatology & Nutrition, The University of Texas MD Anderson Cancer Center, Houston, TX, USA; 2 Department of Genomic Medicine, The University of Texas MD Anderson Cancer Center, Houston, TX, USA; 3 Platform for Innovative Microbiome and Translational Research, The University of Texas MD Anderson Cancer Center, Houston, TX, USA; 4 Department of Surgical Oncology, The University of Texas MD Anderson Cancer Center, Houston, TX, USA; 5 Department of Immunology, The University of Texas MD Anderson Cancer Center, Houston, TX, USA

## Abstract

Immunotherapy has greatly improved cancer outcomes, yet variability in response and off-target tissue damage can occur with these treatments, including immune checkpoint inhibitors (ICIs). Multiple lines of evidence indicate the host microbiome influences ICI response and risk of immune-related adverse events (irAEs). As the microbiome is modifiable, these advances indicate the potential to manipulate microbiome components to increase ICI success. We discuss microbiome features associated with ICI response, with focus on bacterial taxa and potential immune mechanisms involved in irAEs, and the overall goal of driving novel approaches to manipulate the microbiome to improve ICI efficacy while avoiding irAE risk.

## Teaser

The microbiome influences response to immunotherapy, including immune-related adverse events (irAEs) with checkpoint inhibitors. Wang, Jenq, et al. discuss microbiome contributions to irAEs by focusing on major bacterial taxa, potential immune mechanisms, and clinical opportunities for microbiome modulation to treat irAEs.

## Introduction

The microbiome is a key component of human physiology, encompassing microbial organisms in tissues such as the gastrointestinal (GI) tract, skin, and lung (Dethlefsen et al., 2007; Ley et al., 2006). Many microbes have mutualistic relationships with the host and are critical for proper development and function of barrier sites (Bäckhed et al., 2005; Hooper and Macpherson, 2010). The microbiome contributes to health status and disorders such as cancer, obesity, and inflammatory conditions, and influences host responses to therapeutic interventions (Cryan and Dinan, 2012; Gopalakrishnan et al., 2018a; Maeda and Takeda, 2019; Rajilić-Stojanović et al., 2015; Routy et al., 2018a; Turnbaugh et al., 2008). This has been demonstrated amply for cancer, in which the microbiome affects response to immunotherapy, chemotherapy, radiation treatment, and stem cell transplantation (Abu-Sbeih et al., 2019; Chang et al., 2021b; Derosa et al., 2018; Faith et al., 2011; Muegge et al., 2011; Shono et al., 2016; Spencer et al., 2021; Taur et al., 2014; Tonneau et al., 2021; Vétizou et al., 2015; Viaud et al., 2013; Wang et al., 2018c). As the microbiome is modifiable, and likely more amenable to alteration compared to genetic changes driving malignancy, potential to manipulate it to improve cancer outcomes has spurred interest in understanding microbiome–host interactions, their effects on tumor growth, and interplay with cancer treatment.

Immunotherapy is the most significant advance in cancer care in decades, redirecting immune responses to affect durable tumor control. Immune checkpoint inhibitors (ICIs) target negative regulatory proteins and thereby prolong anti-tumor immune responses (Wei et al., 2018). FDA-approved treatments include therapeutic antibodies against cytotoxic T-lymphocyte antigen-4 (CTLA-4), programed cell death protein 1 (PD-1), and PD-L1 (ligand for PD-1), which can be used as monotherapies or in combination with one another or other treatments. While ICI generates long-term control of primary and metastatic disease in many patients, some individuals and cancer types are non-responsive, and tractable approaches to improve ICI efficacy are needed (LaFleur et al., 2018; Ott et al., 2013; Sharma et al., 2021). Manipulation of the fecal microbiome has been shown to convert ICI non-responsive individuals to responsiveness (Baruch et al., 2021b; Davar et al., 2021; Dizman et al., 2022), underscoring potential for microbiome-mediated approaches to potentiate ICI efficacy.

As with other cancer treatments, immunotherapy can drive off-target tissue damage, termed immune-related adverse events (irAEs). The irAEs arise unpredictably and may localize to one or more organs, leading to additional morbidity, halted immunotherapy, and in rare cases death. Approximately 20–60% of ICI-treated individuals experience severe irAEs (grade 3–5), with the incidence varying across treatment regimens (Morad et al., 2022; Wang et al., 2018a). Autoimmune and autoinflammatory responses have been implicated as irAE-driving mechanisms (Kuchroo et al., 2021; Morad et al., 2022; Sullivan and Weber, 2022). Moreover, evidence indicates microbiome effects on irAEs, particularly at barrier sites such as the GI tract and skin (Hu et al., 2022; McCulloch et al., 2022; Morad et al., 2022; Park et al., 2022; Zhou et al., 2023). Here, we discuss the microbiome influence on ICI efficacy and dive more deeply into developing links with irAEs, with the goal of spurring new research and clinical strategies to improve ICI outcomes.

## Microbiome associations with ICI efficacy

The intestinal or fecal microbiome was linked originally with ICI response, while microbiomes in other tissues including skin, lung, or tumors also affect cancer or ICI outcomes (Chaput et al., 2017; Gopalakrishnan et al., 2018b; Iida et al., 2013; McLean et al., 2022; Riquelme et al., 2019; Viaud et al., 2013; Vitorino et al., 2022). In the fecal microbiome, the diversity or species richness of bacteria associates with favorable responses in ICI-treated cancer patients (Chaput et al., 2017; Gopalakrishnan et al., 2018b; Zheng et al., 2019). Consistently, broad-spectrum antibiotic treatment renders poor therapeutic response (Routy et al., 2018b; Wilson et al., 2020). Microbiome profiling by 16S ribosomal RNA gene sequencing or metagenomic analyses reveals associations between specific bacterial taxa and ICI responsiveness including members of the Bacillota phylum (Firmicutes), such as Lachnospiraceae, *Ruminococcus* spp., and *Faecalibacterium* spp.; the Bacteroidota phylum (Bacteroidetes) such as specific *Bacteroides* spp.; the Actinomycetota phylum (Actinobacteria), including *Bifidobacterium* spp. and *Collinsella aerofaciens;* and the Verrucomicrobiota phylum member *Akkermansia muciniphila* (Andrews et al., 2021; Chaput et al., 2017; Derosa et al., 2022; Frankel et al., 2017; Gopalakrishnan et al., 2018b; Hakozaki et al., 2020; Matson et al., 2018; McCulloch et al., 2022; Routy et al., 2018b; Vétizou et al., 2015; Zheng et al., 2019).

ICI non-responsiveness or shorter progression-free survival associates with distinct taxa including Bacillota members *Lactobacillus* spp. and *Streptococcaceae* spp., Bacteroidaceae, and members of the Pseudomonadota phylum (Proteobacteria) such as *Enterobacter* spp. and *Klebsiella* spp. (Andrews et al., 2021; McCulloch et al., 2022; Simpson et al., 2022). Analysis of multiple patient cohorts from discrete geographical locations indicates stronger associations of specific taxa with ICI non-responsiveness versus response (McCulloch et al., 2022). Moreover, poor ICI response correlates with reduced fiber and omega 3 fatty acid intake, which associate with metabolic shifts in the fecal microbiome (Simpson et al., 2022).

**Figure 1.**
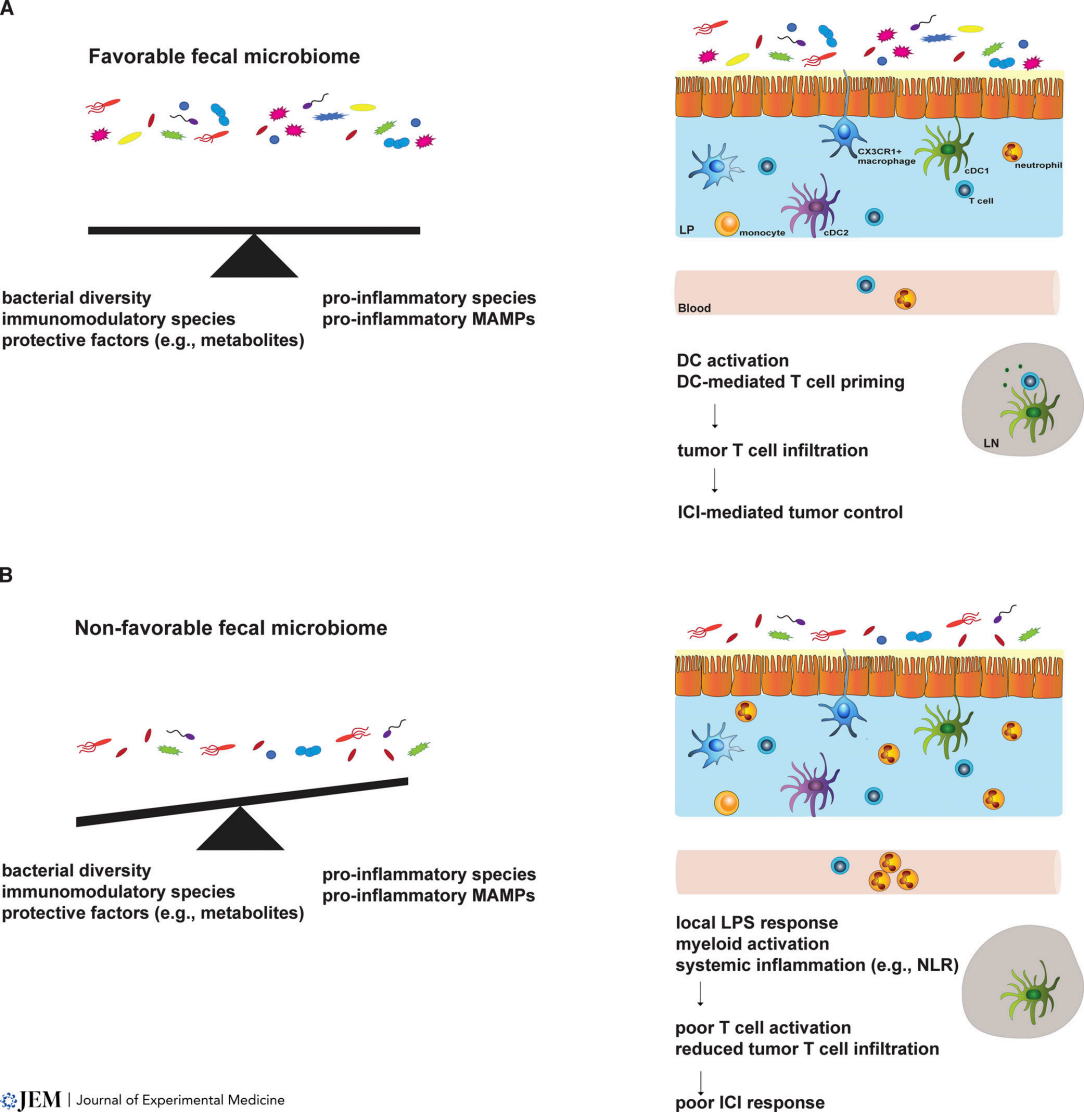
**Fecal microbiome associations with ICI response. (A)** Fecal microbiomes linked with response to ICI show greater diversity, which is predicted to provide an appropriate balance between protective and inflammatory species in the gut (left). Favorable microbiomes associate with increased DC and T cell activation in the intestinal lamina propria (LP), lymph nodes (LN), and tumor (not shown), suggesting they promote effective T cell priming and activation in LNs that enables or enhances ICI response (right). **(B)** Non-favorable microbiomes show less diversity, which is predicted to promote a pro-inflammatory state and reduce protective factors in the gut (left). Non-favorable microbiomes associate with elevated LPS signatures, greater activation of myeloid responses, and increases in circulating neutrophil:lymphocyte ratios (NLR), suggesting microbiome-driven effects on the myeloid compartment interferes with ICI efficacy (right).

To support the possibility that the microbiome can be causally associated with ICI response and unravel cross-talk with the immune system, investigators have pursued mechanistic approaches using transfer of individual bacterial taxa, defined consortia of bacteria, or fecal material from ICI-responder or -non-responder patients to germ-free or antibiotic-treated mice (Mager et al., 2020; Matson et al., 2018; Routy et al., 2018b; Sivan et al., 2015; Spencer et al., 2021; Tanoue et al., 2019). These studies found improved activation of antigen-presenting dendritic cells (DCs) and enhanced tumor infiltration of CD4^+^ and CD8^+^ T cells in the context of favorable taxa or microbiomes (Mager et al., 2020; Matson et al., 2018; Routy et al., 2018b; Sivan et al., 2015; Tanoue et al., 2019) or, conversely, reduced proportions of IFN-γ–positive CD8^+^ T cells in tumors linked with non-favorable fecal microbiomes (Spencer et al., 2021). These immune features align with association between ICI efficacy, DC activation, and tumor T cell infiltration (Cohen et al., 2022; Salmon et al., 2016; Spranger et al., 2017; Spranger et al., 2016). Furthermore, systemic and local (gut) LPS-mediated inflammation and specific metabolic features associate with poor ICI response (McCulloch et al., 2022; Simpson et al., 2022). Collectively, the results to date suggest diverse and favorable microbiomes elicit discrete inflammatory and metabolic responses that improve ICI outcomes, compared to less diverse, non-favorable microbiomes (McCulloch et al., 2022; Simpson et al., 2022; Fig. 1).

Recent clinical trials indicated the potential for microbiome modulation to favorably affect ICI response. Two trials employed fecal microbiota transplantation (FMT) of material obtained from complete ICI responders, transferred to individuals previously non-responsive to therapy, who were restarted on ICI (Baruch et al., 2021b; Davar et al., 2021). Baruch et al. observed clinical responses in 3 out of 10 individuals; notably, all responses occurred in the group of patients receiving FMT from one donor. Davar et al. found clinical benefit in 6 out of 15 individuals following FMT (Davar et al., 2021). Analyses of the local (gut lamina propria), circulating, and tumor immune profiles associated FMT success with enhanced infiltration of CD8^+^ T cells and antigen-presenting cells, similar to findings in pre-clinical models (Baruch et al., 2021b; Davar et al., 2021; Matson et al., 2018; Routy et al., 2018b; Sivan et al., 2015; Fig. 1). FMT facilitated gut microbiome changes in all recipients; however, individuals with subsequent ICI responses showed improved engraftment or stable maintenance of donor microbiomes (Baruch et al., 2021b; Davar et al., 2021), suggesting the ability of “ICI-favorable” microbiota to successfully compete and persist may contribute to FMT success. Separately, a trial employing *Clostridium butyricum*–based supplementation, aimed at enhancing *Bifidobacterium* spp. in the fecal microbiome, suggested enhanced ICI response in renal cell carcinoma (Dizman et al., 2022), supporting the potential for microbiome manipulations to increase ICI efficacy. For additional details on microbiome contributions to ICI response, we refer to expert reviews (Baruch et al., 2021a; Derosa et al., 2021; Fessler et al., 2019; Matson et al., 2021; Morad et al., 2022; Park et al., 2022; Spranger et al., 2016; Zitvogel et al., 2017).

## Microbiome characteristics linked with ICI-related irAEs

The impact of the microbiome on irAEs has been studied most extensively in the context of ICI colitis, where a clear role for bacterial populations was established by evidence that antibiotic treatments increase ICI colitis risk and severity (Abu-Sbeih et al., 2019; Mohiuddin et al., 2021). Moreover, promising clinical findings demonstrate reversal of ICI colitis using FMT in patients refractory to standard-of-care corticosteroids and biologic treatments (Wang et al., 2018c), which implicate the microbiome in mitigating this irAE. Work is also driven by considerable interactions between the gut microbiome and intestinal immune system, as well as the feasibility of sampling the fecal microbiome and intestinal irAE lesions.

ICI colitis is the most frequent irAE with anti-CTLA-4 or anti-PD-1+anti-CTLA-4 (combination ICI) therapy, and can present with the first ICI treatment or as late as greater than 4 mo after completing therapy (Berman et al., 2010; Tian et al., 2018). Approaches to mitigate ICI colitis will have a significant effect on overall irAE burden in cancer patients and potentially enable expansion of current and new ICI treatments to more individuals, including use in neoadjuvant settings.

To diagnose ICI colitis, endoscopic evaluation is recommended with symptoms of grade 2 and higher diarrhea or colitis. These reveal inflammatory lesions in the ileum and/or colon marked by granulocytic infiltration, epithelial apoptosis, cryptitis, disruption of crypt architecture, and other characteristic signs of intestinal inflammation (Tian et al., 2018). Immune profiling of ICI colitis biopsies identified infiltration and activation of cytotoxic T lymphocytes, tissue-resident T cells, and neutrophils, along with increased myeloid cytokines and neutrophil chemoattractants (Hailemichael et al., 2022; Luoma et al., 2020; Zhou et al., 2023). Treatment of ICI colitis depends on the severity, with corticosteroids often used in grade 2 or higher, with or without non-steroid immunomodulators such as blockade of TNF-α (Infliximab) or the gut-homing α4β7 integrin (Vedolizumab; Schneider et al., 2021; Tian et al., 2018).

FMT is a promising approach to treat ICI colitis in patients refractory to standard-of-care therapeutics; FMT also can improve ICI efficacy (Baruch et al., 2021b; Davar et al., 2021; Wang et al., 2018c). Nonetheless, mechanisms by which specific microbiome taxa promote or suppress ICI colitis or other irAEs are largely unknown. To help elucidate this, we focus on specific phyla in the gut microbiome associated with ICI colitis and current understanding of their communication with the immune system, and then discuss host factors and potential new clinical approaches to reduce irAE risk.

## Bacillota

Bacillota members (also termed Firmicutes) are major components of the gut microbiome, comprising Gram-positive organisms including Clostridia, Bacilli, and Mollicutes, which are present in relative greater (Clostridia) or lesser (Bacilli, Mollicutes) abundance (Huttenhower et al., 2012; Ilinskaya et al., 2017; Turnbaugh et al., 2008). Enrichment of Bacillota at the expense of other major phyla, particularly Bacteroidota, correlates with health status versus inflammatory bowel disease (IBD; Miquel et al., 2013; Mukhopadhya et al., 2012; Sokol et al., 2009). By contrast, Bacillota members, including Lachnospiraceae, *Faecalibacterium* spp., *Streptococcus* spp., and *Intestinibacter bartlettii,* have been associated with ICI colitis and other irAEs in independent cohorts of anti-CTLA-4, anti-PD-1, or combination ICI-treated metastatic melanoma patients (Andrews et al., 2021; Chaput et al., 2017; McCulloch et al., 2022). As Lachnospiraceae also correlate with ICI response, McCulloch et al. controlled for time bias and ruled out artefactual association of Lachnospiraceae with elevated risk of irAE development due to increased survival (McCulloch et al., 2022). This approach highlights an important consideration for future microbiome-irAE association studies.

Bacillota express numerous microbial-associated molecular pattern (MAMP) molecules including peptidoglycans, lipoproteins, and unmethylated cytosine–guanine dinucleotide motifs that can stimulate immune reactions. In addition, Bacillota ferment plant polysaccharides to produce short-chain fatty acids (SCFAs; Fernández et al., 2016), which have key roles in the GI tract including serving as fuel for the colonic epithelium, controlling intestinal epithelial barrier function, mucus production, T regulatory (Treg) cell abundance, and restraining inflammatory cytokine production (Siddiqui and Cresci, 2021; Singh et al., 2014). *Faecalibacterium prausnitzii* has also been documented to produce a 15-kD protein with anti-inflammatory properties, while buccal *Megasphaera* spp., which correlate negatively ICI colitis or other irAEs such as pneumonitis, have potential ability to modulate oxidative stress (Nallabelli et al., 2016; Quévrain et al., 2016; Zagato et al., 2020). Thus, Bacillota have diverse capacities to affect intestinal immune responses through activating or suppressive mechanisms, and it remains to be determined whether or how individual taxa directly mediate ICI response or irAEs.

Recent preclinical work has shed light on the contribution of Bacillota to skin irAEs. Hu et al. colonized the skin of mice with *Staphylococcus epidermis*, a commensal that can evade immune activation and exist in a benign relationship with the host (Hu et al., 2022; Otto, 2009). Concurrent treatment with systemic anti-CTLA-4 and *S. epidermis* colonization, however, drove myeloid cell infiltration and elevated inflammatory gene signatures (Hu et al., 2022). Moreover, this combination boosted IFN-γ– and IL-17–producing T cell amounts in skin and skin draining lymph nodes and established an inflammatory T cell memory response that was activated upon *S. epidermis* recolonization alone (Hu et al., 2022). These findings indicate ICI can unleash pathogenic inflammatory responses to a microbiome commensal and provide an elegant mouse model of skin irAEs for further investigation. Interestingly, an analogous commensal-mediated T cell response was identified in allograft rejection (Pirozzolo et al., 2022), underscoring roles for commensals in adverse clinical responses.

## Bacteroidota

Bacteroidota (also termed Bacteroidetes) are Gram-negative anaerobes that comprise a significant proportion of the healthy human fecal microbiome (∼25%). These organisms have been considered “favorable” due to their ability to ferment carbohydrates, produce SCFAs, and associate with a lean body mass. Nonetheless, their transfer to tissues beyond the gut can lead to significant pathologies including bacteriemia (Turnbaugh et al., 2006; Wexler, 2007). Bacteroidota enrichment has been associated with reduced ICI colitis or irAEs in independent studies of metastatic melanoma patients (Chaput et al., 2017; Dubin et al., 2016; Usyk et al., 2021). By contrast, the enrichment of Bacteroidaceae, *Bacteroides dorei*, or *Bacteroides intestinalis* associated with greater irAE risk (Andrews et al., 2021; Simpson et al., 2022; Usyk et al., 2021).

Bacteroidota possess several MAMPs including LPS. Consistently, Bacteroidaceae or *B. intestinalis* abundance associates with host inflammatory factors, including IL-1β (Andrews et al., 2021; Simpson et al., 2022). Moreover, the *B. dorei*–enriched microbiome was characterized by over-representation of adenosine metabolism enzyme capability, suggesting *B. dorei* effects on adenosine catabolism may affect the balance of immunomodulatory and immune-stimulatory responses (Usyk et al., 2021; Vijayan et al., 2017). The LPS of Bacteroidota members, however, is structurally distinct from other Gram-negative bacteria such as *Escherichia coli*, with reported inhibitory properties (Vatanen et al., 2016). Further mechanistic studies are needed to examine links between Bacteroidota MAMPs and metabolic pathways with intestinal and systemic immune responses and irAEs.

## Actinomycetota

Among the most studied taxa in this phylum (also termed Actinobacteria) in the context of ICI response and toxicity are *Bifidobacterium *spp., Gram-positive anaerobes, which are also early colonizers of the intestinal microbiome during the neonatal period, with key roles in establishment of immune subsets in the GI tract (Ruiz et al., 2017). Protective functions for *Bifidobacterium* spp. were identified in an initial model of ICI colitis. Mice treated with dextran sodium sulfate (DSS), which damages the intestinal epithelium, showed exacerbated pathology with CTLA-4 blockade (Wang et al., 2018b). Colonic tissue damage was further enhanced by vancomycin, an antibiotic targeting Gram-positive bacteria, while a probiotic cocktail of *Bifidobacterium* spp. largely abrogated toxicity (Wang et al., 2018b). Moreover, oral transfer of *Bifidobacterium breve* decreased colonic irAE and enhanced Treg functional capacity in the gut. Transfer of *Lactobacillus rhamnosum* (Bacillota), enriched following *Bifidobacterium*-based supplementation, showed similar ability to alleviate DSS-driven irAE (Sun et al., 2020).

In separate studies, *Bifidobacterium adolescentis* was shown to mediate Th17 generation in the small intestine (Ang et al., 2020). Th17 cells can elicit protective or inflammatory responses in the intestinal immune system, and can be regulated by other microbial species including segmented filamentous bacteria (Ivanov et al., 2009; Omenetti et al., 2019). Interestingly, *Bifidobacterium *spp. and Th17 were modulated by a ketogenic diet (Ang et al., 2020). Diet-mediated effects on ICI efficacy and irAE occurrence have been reported (Ang et al., 2020; Lee et al., 2020; Simpson et al., 2022; Spencer et al., 2021; Spyrou et al., 2021), yet much remains to be understood about causal relationships through diet-influenced microbiomes.

## Pseudomonadota

Pseudomonadota members (also termed Proteobacteria) are Gram-negative bacteria with LPS-containing outer membranes. This phylum comprises several pathogens, including deleterious strains of *E. coli*, and its overrepresentation in the intestinal microbiome is linked with IBD (Mukhopadhya et al., 2012). Increased Pseudomonadota abundance in the gut microbiome of metastatic melanoma patients treated with anti-PD-1 also associates with tumor progression or lack of ICI response (McCulloch et al., 2022). Moreover, microbiome profiles characterized by enrichment of Gram-negative bacteria and LPS synthesis genes correlate with poor ICI outcomes (McCulloch et al., 2022; Simpson et al., 2022).

**Figure 2.**
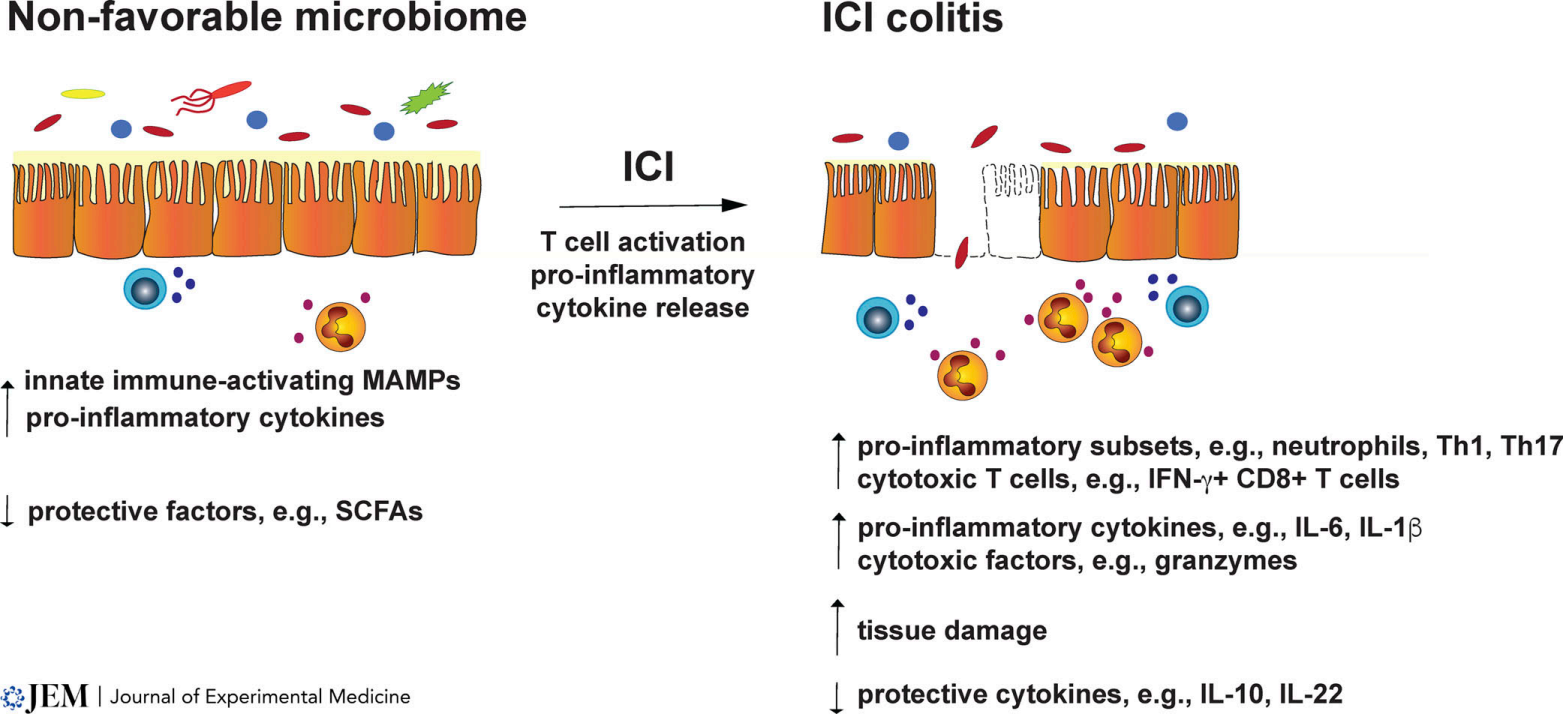
**Model of microbiome-mediated effects on ICI colitis.** Microbiomes associated with increased risk of ICI colitis are predicted to elicit elevated production and/or immune recognition of MAMPs, resulting in increased pro-inflammatory cytokine production from intestinal immune subsets. These microbiomes are also expected to associate with reduced production of gut-protective factors such as SCFAs. These responses lead to induction of a pro-inflammatory state in the gut (left). Upon T cell activation by ICI and consequent production of T cell–produced inflammatory cytokines (e.g., IFN-γ, TNF-α), the intestinal environment is shifted further toward a pro-inflammatory state that drives epithelial barrier disruption, increases exposure to MAMPs, and further promotes localized inflammatory responses that further tissue damage, which drive clinical signs of ICI colitis.

McCulloch and colleagues evaluated intestinal immune responses in ICI-treated individuals through transcriptomic profiling of luminal cells shed in the fecal material (the exfoliome). These studies linked ICI non-responsiveness with signs of intestinal inflammation, including an enhanced LPS-responsive gene signature in the exfoliome, increases in pro-inflammatory cytokine and transcriptional regulator gene expression, and elevated inflammatory cell subsets (DCs, monocytes, macrophages; McCulloch et al., 2022). Poor response to ICI also correlates with increases in circulating neutrophil:lymphocyte ratios or C-reactive protein, suggestive of elevated systemic inflammation (McCulloch et al., 2022; Simpson et al., 2022). In addition, poor ICI outcomes including increased irAE severity or risk associate with a reduction in beneficial metabolic features of the microbiome (Simpson et al., 2022). The collective findings to date suggest microbiomes that predispose to ICI colitis are enriched in Gram-negative and Pseudomonadota members, which induce pro-inflammatory shifts that facilitate tissue damage upon T cell activation by ICI (Fig. 2).

## Host and environmental factors

### Cytokines

Inflammatory cytokines including IL-1β, TNF-α, and IL-6 are linked with effects of the fecal microbiome on ICI colitis; IL-6 also drives irAEs in other organs (Andrews et al., 2021; Dimitriou et al., 2021; Hailemichael et al., 2022; Kim et al., 2017; Perez-Ruiz et al., 2019; Uemura et al., 2016; Zhou et al., 2023). While IL-1β blockade reduced ICI colitis severity in mice, IL-6 inhibition improved tumor response to ICI and suppressed irAE symptoms in preclinical models, and is being pursued as an approach to alleviate irAEs in patients (Andrews et al., 2021; Dimitriou et al., 2021; Hailemichael et al., 2022; Kim et al., 2017; Uemura et al., 2016; Zhou et al., 2023). These inflammatory cytokines are induced by MAMPs, often in myeloid and DC populations, suggesting microbiome-associated factors affect their production by innate immune subsets. Nonetheless, whether or how ICI shapes communication between MAMPs and the host immune system including molecular sensors, cell types, or magnitude of response requires significant work to unravel.

IFN-γ and IL-17, which are generally produced by lymphocytes, are linked with ICI colitis and skin irAEs (Hailemichael et al., 2022; Hu et al., 2022; Zhou et al., 2023). IFN-γ is favorable in the context of ICI and tumor immune responses, yet IFN-γ can promote off-target inflammation. This may be particularly relevant in the gut, as IFN-γ mediates myeloid cell activation, intestinal epithelial cell turnover, and disrupts epithelial barrier function (Bruewer et al., 2003; Cao et al., 2022). Moreover, IL-17 is an inflammatory factor involved in production of myeloid cells that induce tissue damage (Kanai et al., 2012; Xu and Cao, 2010). As IL-6 drives Th17 generation and is one of the most elevated cytokines in human ICI colitis, results to date suggest an IL-6–Th17 axis contributes to irAEs (Hailemichael et al., 2022; Zhou et al., 2023).

By contrast, canonically protective cytokines in the gut associate with favorable microbiome taxa. *Bifidobacterium* spp. mediated IL-10 and IL-22 production in a DSS-driven model of anti-CTLA-4-mediated colitis (Sun et al., 2020). IL-10 also associated with protective effects of *Bacteroides fragilis* on ICI colitis (Vétizou et al., 2015). Moreover, microbiome composition was linked to Tregs, a major IL-10–producing subset (Sun et al., 2020; Vétizou et al., 2015). These findings provide insights into mitigative factors mediated by the microbiome and lay important groundwork for further work to parse additional protective as well as deleterious mechanisms in irAEs.

### Additional host and environmental factors

Autoimmunity or autoinflammatory conditions such as IBD increase irAE risk (Abu-Sbeih et al., 2019; Brown et al., 2021; Grover et al., 2020). As genetic status raises propensity for these disorders, genetic characteristics may impact irAE risk. While this remains to be explored in human cohorts, preclinical studies showed genetically modified mice predisposed to intestinal inflammation develop severe inflammatory responses upon CTLA-4 blockade (Zhou et al., 2023). This work also implicated acute GI infection with the murine pathogen *Citrobacter rodentium* in driving enhanced intestinal tissue damage during anti-CTLA-4 therapy (Zhou et al., 2023), raising the possibility that acquired infections in ICI-treated individuals increase risk for irAEs. Separately, two reports implicated medication use, specifically proton-pump inhibitors, as increased in patients who develop irAEs (Kostine et al., 2021; McCulloch et al., 2022). Understanding host risk factors for irAEs is feasible in preclinical models and appropriately designed clinical studies, and is important for better prediction of ICI outcomes.

Bacterial Ags that mimic tissue Ags and drive tissue inflammation have been identified in diseases such as inflammatory cardiomyopathy or autoimmune diabetes (Gil-Cruz et al., 2019; Girdhar et al., 2022). These findings raise the possibility that commensal-specific T cells, which can favorably affect ICI-mediated tumor control, may elicit autoreactive responses that drive irAEs (Fluckiger et al., 2020; Geuking and Burkhard, 2020; Hayase and Jenq, 2021; Hu et al., 2022; Naqash et al., 2021). This concept is consistent with ICI colitis immune reactions characterized by tissue-resident CD8^+^ T cell populations, and association between peripheral T cell diversity and greater irAE likelihood (Andrews et al., 2021; Luoma et al., 2020). In addition, a recent study of ICI-mediated myocarditis identified α-myosin autoreactive T cells (Axelrod et al., 2022), yet whether the microbiome contributes to this high-risk irAE is unclear.

By contrast, certain bacterial products have immunomodulatory roles that may help explain beneficial effects of individual microbiome taxa. For instance, *A. muciniphila*, which associates with ICI response, produces a diacyl phosphatidylethanolamine recognized by a TLR1–TLR2 heterodimer (Bae et al., 2022). Signaling through this complex results in lowered pro-inflammatory factor production relative to canonical TLR2 agonists, suggesting this modification of the TLR2 response as a mechanism of immune modulation by *A. muciniphila* (Bae et al., 2022). In addition, Bacteroidota members produce β-hexosaminidase, which supports the differentiation of intraepithelial CD4^+^ lymphocytes that have protective roles against intestinal inflammation (Bousbaine et al., 2022). Additional protective metabolic factors, such as SCFAs, also result from microbial activity in the gut (Fernández et al., 2016; Turnbaugh et al., 2006; Wexler, 2007). Future work may help identify microbiome products that could serve as potential targets for therapeutic development to suppress ICI colitis or other irAEs.

## Need for additional mechanistic studies

A greater understanding of MAMPs, metabolites, Ags, and other products produced by microbiome species, along with how this production is affected by microbiome community composition, and how these factors regulate host immune responses, is needed to elucidate microbiome-driven irAE mechanisms. Future studies must consider the specific immune environment in an irAE target tissue (e.g., skin, intestine) and aim to establish mechanistic links with host components such as sensor molecules (e.g., TLRs), soluble factors (e.g., cytokines), and specific immune and non-immune populations. This work can be informed through studies that delineated innate immune responses against isolated gut bacterial strains from healthy individuals and those with IBD, which found variations across taxa and specific host sensing mechanisms (e.g., TLRs; Spindler et al., 2022). Use of genetically engineered or humanized mice, FMT with bacterial consortia or patient material, and narrow-spectrum antibiotics will also help elucidate the contribution of specific bacterial taxa to ICI colitis and other irAEs (Cheng et al., 2022; Maslowski, 2019; Tanoue et al., 2019; Andrews et al., 2021; Hailemichael et al., 2022; Zhou et al., 2023).

## Clinical approaches and additional opportunities

### Treatment of ICI colitis by FMT

For ICI colitis treatment, FMT need not be limited to particular types of cancers, however strict patient selection (e.g., avoidance of neutropenic individuals) is critical to ensure lower risk of complication. In experimental colitis, FMT is associated with alterations in the microbiome composition, shifts in the balance of immune-activating and regulatory populations in the gut, and differential pro- and anti-inflammatory cytokine production (Burrello et al., 2018). Stool biomarkers such as calprotectin can help guide clinical decisions regarding treatment duration and predict colitis remission (Zou et al., 2021). Nonetheless, much remains to be learned regarding clinical features that enable FMT success. To help guide future work, we outline outstanding questions (see text box). These can be addressed by further clinical study, translational work utilizing preclinical models with patient material, and new mechanistic investigations.

**Outstanding questions related to clinical success of FMT in ICI colitis**

**(1) Which factors predict the clinical response of a patient with ICI colitis to FMT?**

(a) Microbiome composition prior to or following FMT?

(b) Host immune status prior to FMT?

(c) Clinical or histological factors?

(d) Other?

**(2) Which characteristics of FMT donors are most important for clinical response?**

(a) Are there unique features of FMT donors that drive FMT success?

(b) Alternatively, will most healthy individuals equally suffice as FMT donors?

**(3) How does FMT regulate the microbiome and how is this linked to clinical efficacy?**

(a) Does FMT function primarily by introducing beneficial, immune-regulatory bacteria?

(b) Does FMT largely operate by suppressing existing inflammatory bacteria?

(c) Alternatively, are both activities (introduction of beneficial species and suppression of inflammatory species) required for the clinical efficacy of FMT in ICI colitis?

**(4) Which host responses are affected by FMT and how do these impact clinical response?**

(a) Does FMT function by modifying the activity or abundance of intestinal immune cell populations?

(b) Are other cell populations in the GI tract involved in FMT response, including the intestinal epithelium?

(c) Does FMT affect mucus protection, anti-microbial peptide production, or cytokine release in the GI tract?

(d) Are other features of the GI tract involved in the clinical response to FMT?

(e) Does FMT impact the systemic immune and subsequent ICI responses?

(f) Other?

## Next steps for advancement of clinical trials

Numerous clinical trials are underway to evaluate FMT in management of diagnosed ICI colitis and/or for improvement of ICI efficacy (Table 1). It remains an open question as to whether FMT should be provided prior to or after the onset of ICI colitis. In considering trials to evaluate FMT as a frontline therapy, as opposed to steroids or biologics, focusing on cancer types that are frequently treated with ICI and shown to respond to FMT (e.g., melanoma) may facilitate patient enrollment. Moreover, a standardized endoscopic scoring system would be beneficial. Similarly, whether other microbiome manipulations would be effective before individuals are started on ICI therapy or during treatment is unknown. As investigations reveal microbiome signatures that contribute to ICI response, ICI colitis, or other irAEs, it may become possible to explore options that alter the microbiome prior to or at the time of ICI initiation to improve response and/or lower the risk of toxicity. To advance the field, clinical trials that use interventional approaches and randomized studies are preferred, although this can be challenging if enrollment is difficult.

**Table 1.** Clinical trials to evaluate the efficacy of FMT in ICI response and irAE risk (top) or ICI colitis treatment (bottom)

ClinicalTrials.gov identifier	Study title	Condition or Disease	Intervention/Treatment (non-ICI)	Phase and status
Clinical trials for cancer				
NCT05008861	Gut microbiota reconstruction for NSCLC immunotherapy	Non-small cell lung cancer	FMT Platinum-based chemotherapy	Phase 1 Not yet recruiting
NCT04729322	Fecal microbiota transplant and re-introduction of anti-PD-1 therapy (Pembrolizumab or Nivolumab) for the treatment of metastatic colorectal cancer in anti-PD-1 non-responders	Colorectal cancer Small intestinal adenocarcinoma	FMT Metronidazole Neomycin Vancomycin	Early Phase 1 Recruiting
NCT04163289	Preventing toxicity in renal cancer patients treated with immunotherapy using fecal microbiota transplantation (PERFORM)	Renal cell carcinoma	FMT	Phase 1 Recruiting
NCT04130763	Fecal microbiota transplant (FMT) capsule for improving the efficacy of anti-PD-1	Gastrointestinal system cancer	FMT	Phase 1 Unknown
NCT05502913	Fecal microbiota transplantation with immune checkpoint inhibitors in lung cancer	Metastatic lung cancer	FMT Antibiotics	Phase 2 Not yet recruiting
NCT05279677	FMT combined with immune checkpoint inhibitor and TKI in the treatment of CRC patients with advanced cancer	Colorectal neoplasms, malignant	FMT	Phase 2 Recruiting
NCT05251389	FMT to convert response to immunotherapy	Melanoma Stage III and IV	FMT	Phase 1 Phase 2 Recruiting
NCT04758507	Fecal microbiota transplantation to improve efficacy of immune checkpoint inhibitors in renal cell carcinoma (TACITO)	Renal cell carcinoma	FMT	Phase 1 Phase 2 Recruiting
NCT05273255	Fecal microbiota transplantation in patients with malignancies not responding to immune checkpoint inhibitor therapy	Any cancer	FMT	Not applicable Recruiting
NCT04577729	The IRMI-FMT trial	Malignant melanoma, stage III or IV	FMT, allogeneic FMT, autologous	Not applicable Recruiting
Clinical trials for ICI colitis				
NCT04038619	Fecal microbiota transplantation in treating immune-checkpoint inhibitor induced-diarrhea or colitis in genitourinary cancer patients	Malignant genitourinary system neoplasms ICI colitis Diarrhea	FMT Loperamide	Phase 1 Recruiting
NCT04883762	Stool transplant to control treatment-related diarrhea	FMT ICI colitis	FMT	Phase 1 Recruiting
NCT03819296	Role of gut microbiome and fecal transplant on medication-induced GI complications in patients with cancer	Solid tumors ICI colitis	FMT Infliximab Prednisone Vedolizumab	Phase 1 Phase 2 Recruiting

Furthermore, as clinical studies unfold, it is key to account for prior antibiotic use, concurrent medications, or other factors including diet that have potential to affect the microbiome or host immune status. In addition, microbiome therapies such as FMT may benefit from approaches to improve safe and effective engraftment such as supplementation with prebiotics to support growth of beneficial species (Button et al., 2022; Chang et al., 2021a; Hayase et al., 2022). Moreover, novel targeting approaches such as lytic bacteriophages or CRISPR-based methods may be useful for removing key taxa driving ICI non-responsiveness or irAEs, as indicated by recent advancements in phage-directed targeting of IBD-associated *Klebsiella pneumoniae* that reduces intestinal inflammation in animal studies (Federici et al., 2022).

In addition to the above, we must also consider the microbiome as a functional network comprised not only of bacteria, but also other microbes such as fungi, archaea, viruses, and protozoa (Falony et al., 2016). To capture this complexity, we must embrace more holistic assessments of the microbiome through metagenomic sequencing, metabolomic profiling, and other approaches. We anticipate significant developments in understanding microbiome-mediated mechanisms that affect ICI outcomes as new studies include analyses of discrete bacterial species, non-bacterial components, the composition of microbiomes from multiple tissues, host immune responses, and assessment of individuals from distinct geographic locations. We also hope to see advancement of ICI and other immunotherapies reaching broader patient populations and expect this will bring further growth in our understanding of microbiome-mediated effects on health, disease, and response to therapy.
